# Development and validation of a nomogram for predicting recurrent plastic bronchitis in children with Mycoplasma pneumoniae pneumonia

**DOI:** 10.3389/fped.2026.1959849

**Published:** 2026-09-09

**Authors:** Feiyang Na, WanYi Li, Qijun Zhao, Bohan Ma, Yannan Wang, Li Zhou, Zhizhen Chen, Hongyu Zhang, Junlin Zhou, Jianli Liu

**Affiliations:** 1 Department of Radiology, the Second Hospital & Clinical Medical School, Lanzhou University, Lanzhou, China; 2 Department of Pediatric, Gansu Provincial Maternal and Child-care Hospital (Gansu Provincial Central Hospital), Lanzhou, China; 3 Faculty of Data Science, City University of Macau, Macao, Macao SAR, China

**Keywords:** children, Mycoplasma pneumoniae pneumonia, nomogram, prediction model, recurrent plastic bronchitis

## Abstract

**Background:**

Plastic bronchitis (PB) is a complication of Mycoplasma pneumoniae pneumonia (MPP) in children, characterized by bronchial cast formation and airway obstruction. Recurrence after bronchoscopic cast removal can occur, and tools for early risk prediction are limiting. This study aimed to develop and externally validate a nomogram for predicting recurrent PB in children with MPP after initial bronchoscopic cast removal.

**Methods:**

This retrospective multicenter cohort study included pediatric patients with MPP complicated by PB. Patients from one center were randomly divided into a training cohort and an internal validation cohort at a ratio of 8:2, while an independent cohort from another tertiary hospital served as the external validation chort. Candidate predictors were screened using least absolute shrinkage and selection operator (LASSO) regression, followed by multivariable logistic regression to identify independent predictors and develop the prediction model. Model performance was evaluated by discrimination, calibration, and clinical utility using receiver operating characteristic (ROC) curves, calibration plots, the Hosmer-Lemeshow goodness-of-fit test, and decision curve analysis (DCA).

**Results:**

A total of 352 children with MPP complicated by PB were included, with 281 patients in the training cohort and 71 in the internal validation cohort. An additional 44 patients from an independent center formed the external validation cohort. Four variables were identified as independent predictors of PB recurrence: serum albumin (ALB), fever persisting for ≥72 h after the first bronchoalveolar lavage (BAL), atelectasis, and bronchial casts involving ≥2 lung lobes. These predictors were incorporated into a nomogram. The model showed discrimination across the three cohorts, with an area under curve (AUC) of 0.853 in the training cohort, 0.833 in the internal validation cohort, and 0.811 in the external validation cohort. Calibration plots showed agreement between predicted and observed risks, and decision curve analysis indicated a higher net benefit than the treat-all and treat-none strategies across a range of threshold probabilities.

**Conclusion:**

We developed and externally validated a nomogram to predict PB recurrence in children with MPP using routinely available clinical and bronchoscopic variables. The model showed consistent discrimination and calibration. Its potential clinical utility for risk stratification and post-procedural monitoring requires further prospective evaluation.

## Introduction

Mycoplasma pneumoniae pneumonia (MPP) remains a major cause of community-acquired pneumonia (CAP) in children and presents with a broad clinical spectrum, ranging from mild, self-limiting illness to rapidly progressive disease with extensive airway involvement (1–3). In a subset of patients, severe airway inflammation and epithelial injury can lead to excessive inflammatory exudation, impaired mucociliary clearance, and accumulation of highly viscous secretions, ultimately resulting in plastic bronchitis (PB). PB is a serious complication characterized by the formation of bronchial casts that obstruct the airway and may cause hypoxemia, atelectasis, and progressive radiographic abnormalities (4).

Bronchoscopy with bronchoalveolar lavage (BAL) and bronchial cast extraction is widely used to relieve airway obstruction and restore ventilation in patients with PB (5). However, clinical practice suggests that airway clearance may not always resolve the underlying disease process. Despite initially successful intervention, some children experience recurrent cast formation, requiring repeated bronchoscopic procedures and prolonged hospitalization. Recurrent PB poses a substantial clinical challenge, as it increases procedural exposure and healthcare utilization and may contribute to persistent airway obstruction and radiographic abnormalities. Moreover, the optimal timing for clinical reassessment and repeat bronchoscopic intervention remains uncertain.

To address these challenges, early identification of children at high risk of PB recurrence after initial BAL may enable tailored monitoring and facilitate timely escalation of airway interventions. However, existing studies have largely focused on development of PB itself or have combined PB cases with heterogeneous etiologies, limiting their applicability to children with MPP-associated PB (6–8). Moreover, potential predictors have often been evaluated individually, and few predictive tools have integrated systemic inflammatory markers, imaging evidence of airway obstruction, and initial bronchoscopic findings into an individualized and clinically interpretable risk prediction model.

In this study, we retrospectively analyzed clinical data related to children with MPP complicated by PB, developed a nomogram to predict the risk of recurrence using routinely available clinical, radiological, and initial bronchoscopic findings, and externally validated the model. By providing an individualized estimate of recurrence risk, this model may facilitate early identification of children at high risk of recurrent PB, support personalized post-procedural monitoring, and assist clinicians in making timely decisions regarding repeat bronchoscopy.

## Materials and methods

The model was developed and reported in accordance with the Transparent Reporting of a Multivariable Prediction Model for Individual Prognosis or Diagnosis (TRIPOD) statement (9, 10). This study was approved by the Institutional Review Board of Gansu Provincial Maternity and Child-Care Hospital (Approval No. 2023GSFY-57) and the Ethics Committee of Lanzhou University Second Hospital (Approval No. 2025A-831). All procedures were conducted in accordance with applicable ethical guidelines and regulations. Written informed consent was waived because of the retrospective design.

### Participants

We retrospectively and consecutively included pediatric patients with MPP complicated by PB who underwent bronchoscopy at Gansu Provincial Maternity and Child-Care Hospital (Gansu Provincial Central Hospital) between July 2023 and January 2026 and at Lanzhou University Second Hospital between December 2024 and January 2026. Eligible patients were identified from electronic medical records. The inclusion criteria were as follows: (1) aged 1 month to 18 years; (2) MPP diagnosed according to the 2023 Chinese guideline (11); and (3) PB confirmed by bronchoscopy, defined as the presence of extensive or localized rubbery casts in the trachea or bronchi that were removed during the procedure. The exclusion criteria were as follows: (1) incomplete clinical data; (2) congenital heart disease (CHD) or a history of cardiac surgery; (3) confirmed abnormalities in lymphatic drainage; and (4) coinfection with other pathogens.

### Data collection

The following variables were extracted from electronic medical records: (1) demographic and clinical characteristics, including sex, age, duration of fever, peak temperature, cough, dyspnea, and hypoxemia; (2) laboratory indices, including white blood cell count (WBC), platelet count (PLT), C-reactive protein (CRP), procalcitonin (PCT), alanine aminotransferase (ALT), aspartate aminotransferase (AST), lactate dehydrogenase (LDH), creatine kinase (CK), creatine kinase-MB (CK-MB), albumin (ALB), and D-dimer; (3) treatment-related variables, including time from symptom onset to the first bronchoscopy, fever persisting for ≥72 h after first BAL, mechanical ventilation, intravenous corticosteroid therapy, intravenous immunoglobulin (IVIG), and replacement of macrolides with tetracyclines or fluoroquinolones before BAL; (4) chest imaging findings, including atelectasis, lobar consolidation involving ≥2/3 of a lobe, pleural effusion, and pulmonary lesions involving ≥2 lobes; and (5) initial bronchoscopic findings, including bronchial casts involving ≥2 lung lobes, bilateral lung involvement, central airway involvement and severe airway mucosal injury.

### Initial bronchoscopic findings

Initial bronchoscopic findings were assessed during the first flexible bronchoscopy after admission. Bronchoscopy reports and stored images and videos were reviewed, and the location, distribution, and extent of plastic casts were recorded. Bronchial casts involving ≥2 lung lobes were defined as visible casts obstructing segmental or subsegmental bronchi in at least two different lobes. Bilateral lung involvement was defined as the presence of plastic casts in both lungs, whereas central airway involvement referred to casts extending into the trachea and/or main bronchi. Severe airway mucosal injury was defined as bronchial mucosal erosion and/or ulceration observed during the initial bronchoscopy. Mucosal congestion, edema, or contact bleeding alone was not considered severe airway mucosal injury.

### Radiographic assessment

All chest imaging examinations were independently reviewed by two radiologists with 6 and 10 years of experience, respectively, in pediatric thoracic imaging. In cases of disagreement, a third senior radiologist with 15 years of experience in pediatric thoracic imaging adjudicated the final assessment. Atelectasis was defined as partial or complete collapse of a pulmonary segment or lobe, accompanied by volume loss and increased pulmonary opacity. Lobar consolidation involving ≥2/3 of a lobe was defined as consolidation occupying at least two-thirds of a single pulmonary lobe. Pleural effusion was defined as the presence of fluid within the pleural space. Lesions involving ≥2 lobes were defined as pulmonary lesions affecting two or more pulmonary lobes.

### Outcomes

The primary outcome was PB recurrence during the same disease course. Patients were categorized into a single-episode PB group and a recurrent PB group according to the number of PB episodes. At both participating centers, similar clinical indications were routinely used to guide repeat bronchoscopy. Specifically, repeat bronchoscopy was considered in children with persistent or worsening respiratory symptoms, persistent fever and/or an inadequate clinical response following the initial bronchoscopic intervention and subsequent treatment, or lack of expected radiological improvement (12). The final decision to perform repeat bronchoscopy was made by the treating clinical team based on an integrated assessment of the patient's clinical course and imaging findings. The recurrent PB group comprised patients whose bronchial casts had been completely removed during the initial bronchoscopy and in whom newly formed bronchial casts were subsequently confirmed by repeat bronchoscopy.

### Statistical analysis

Patients from Gansu Provincial Maternity and Child-Care Hospital (Gansu Provincial Central Hospital) were randomly divided into a training cohort and an internal validation cohort at a ratio of 8:2 for model development and internal validation. External validation was subsequently performed using an independent dataset from Lanzhou University Second Hospital.

Missing data were observed only for peak temperature, with 7 (2.49%), 4 (5.63%), and 3 (6.82%) missing values in the training, internal validation, and external validation cohorts, respectively. Missing values were imputed using multiple imputation by chained equations (MICE). No missing data were observed for the remaining variables. Details of the missing-data pattern are provided in Supplementary Table 1.

The normality of continuous variables was assessed using the Shapiro–Wilk test. Normally distributed continuous variables are presented as mean ± standard deviation (SD), whereas non-normally distributed continuous variables are presented as median [interquartile range (IQR)]. Categorical variables are reported as counts and percentages. For comparisons between two groups, categorical variables were compared using the chi-square test or Fisher's exact test, as appropriate, whereas continuous variables were compared using the independent-samples t-test or Mann–Whitney U test, as appropriate. For overall comparisons among the training, internal validation, and external validation cohorts, normally distributed continuous variables were compared using one-way analysis of variance (ANOVA), non-normally distributed continuous variables using the Kruskal–Wallis H test, and categorical variables using the chi-square test or Fisher's exact test, as appropriate.

Interobserver agreement between the two primary radiologists regarding chest CT findings was assessed using unweighted Cohen's kappa (*κ*) coefficients with 95% confidence intervals (CIs). Kappa coefficients were calculated based on the radiologists’ initial independent assessments before adjudication by the third senior radiologist.

LASSO regression was performed exclusively in the training cohort to screen candidate predictors and reduce model complexity. The penalty parameter (*λ*) was determined using five-fold cross-validation, and the optimal *λ* was selected according to the one-standard-error criterion (*λ*1se). Variables retaining nonzero coefficients at the selected *λ* were subsequently entered into a multivariable logistic regression model. In the training cohort, 85 recurrent PB events were observed, and six variables selected by LASSO were entered into the multivariable logistic regression model, corresponding to an events-per-variable (EPV) ratio of 14.17 (85/6). A nomogram was then constructed based on the predictors retained in the final multivariable model to facilitate individualized risk estimation.

Model discrimination was evaluated using receiver operating characteristic (ROC) curves and quantified by the area under the curve (AUC) with 95% CIs. Calibration was assessed using calibration plots and the Hosmer-Lemeshow goodness-of-fit test. Clinical utility was evaluated using decision curve analysis (DCA) to estimate the net benefit across a range of threshold probabilities. All statistical tests were two-sided, and a *P* value of < 0.05 was considered statistically significant. All statistical analyses were performed using IBM SPSS Statistics version 22.0 and R software version 4.4.0.

## Results

### Subject characteristics

**Figure 1:**
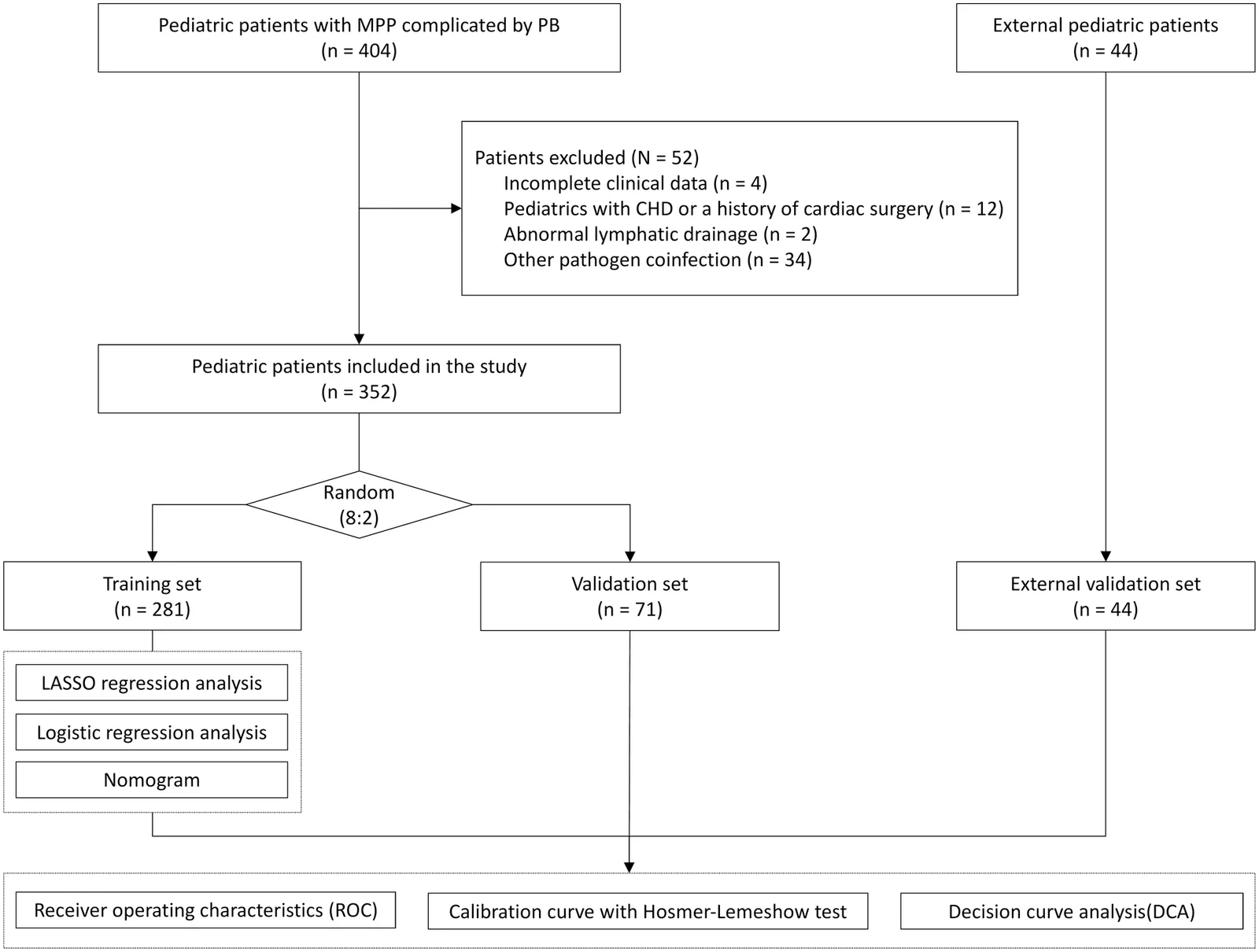
Flowchart of patient recruitment and allocation.

A total of 352 pediatric patients with MPP complicated by PB were included in this study and were randomly assigned to a training cohort (*n* = 281) and an internal validation cohort (*n* = 71). The external validation cohort consisted of 44 patients from the Lanzhou University Second Hospital. The study flowchart is presented in Figure 1.

In the training cohort, 196 patients were classified into the single-episode PB group and 85 into the recurrent PB group. The baseline characteristics of the two groups are summarized in Table 1. Compared with patients in the single-episode PB group, those in the recurrent PB group had a significantly higher peak temperature and higher levels of CRP, PCT, ALT, AST, LDH, CK-MB, and D-dimer, as well as lower ALB levels. In addition, fever persisting for ≥72 h after the first BAL, intravenous corticosteroid therapy, atelectasis, lobar consolidation involving ≥2/3 of a lobe, pleural effusion, and bronchial casts involving ≥2 lung lobes were more frequently observed in the recurrent PB group (all *P* < 0.05). The baseline characteristics did not differ significantly among the training, internal validation, and external validation cohorts (all *P* > 0.05; Supplementary Table 1). Interobserver agreement for chest CT and initial bronchoscopic findings was excellent, with all estimable Cohen's *κ* values exceeding 0.90 (Table 2).

**Table 1:** The parameters of the derivation cohort were compared between single PB group and recurrent PB group.

Variables	Single PB group	Recurrent PB group	*P* value
Patients, No.	196	85	
Clinical characteristics			
Sex, *n* (%)			0.950
Male	99 (50.51)	44 (51.76)	
Female	97 (49.49)	41 (48.24)	
Age, years	7.82 ± 2.37	7.30 ± 2.83	0.137
Duration of fever, days	8.00 (6.75,9.50)	8.50 (7.00,10.00)	0.186
Fever, *n* (%)			—
No	0 (0.00)	0 (0.00)	
Yes	196 (100)	85 (100.00)	
Peak temperature, °C	39.60 (39.20,40.00)	40.00 (39.50,40.00)	0.003
Cough, *n* (%)			—
No	0 (0.00)	0 (0.00)	
Yes	196 (100)	85 (100.00)	
Dyspnea, *n* (%)			0.132
No	183 (93.37)	74 (87.06)	
Yes	13 (6.63)	11 (12.94)	
Hypoxemia, *n* (%)			0.087
No	179 (91.33)	71 (83.53)	
Yes	17 (8.67)	14 (16.47)	
Laboratory tests			
WBC, 10^9^/L	7.36 (6.03,9.36)	7.99 (5.97,9.51)	0.413
PLT, 10^9^/L	250.00 (207.00,302.00)	274.00 (229.00,328.25)	0.079
CRP, mg/L	19.58 (11.14,33.80)	31.72 (14.32,54.58)	0.002
PCT, ng/mL	0.22 (0.14,0.35)	0.30 (0.18,0.68)	0.005
ALT, U/L	13.50 (10.30,20.68)	22.60 (14.40,39.00)	<0.001
AST, U/L	31.65 (24.50,46.70)	41.90 (31.50,54.90)	<0.001
LDH, U/L	339.50 (262.75,452.00)	480.00 (397.00,685.00)	<0.001
CK, U/L	92.50 (58.00,155.25)	94.00 (63.00,227.00)	0.444
CK-MB, U/L	21.20 (16.38,33.30)	25.90 (19.30,38.40)	0.011
ALB, g/L	42.61 (5.34)	37.09 (4.87)	<0.001
D-dimer, mg/L	0.93 (0.49,1.81)	2.05 (1.15,4.09)	<0.001
Treatment characteristics			
Time from onset to the first bronchoscopy,days	9.00 (7.00,10.00)	8.00 (7.00,10.00)	0.268
Fever persisting for≥72 h after the first BAL, *n* (%)			<0.001
No	189 (96.43)	64 (75.29)	
Yes	7 (3.57)	21 (24.71)	
Mechanical ventilation, *n* (%)			0.204
No	193 (98.47)	81 (95.29)	
Yes	3 (1.53)	4 (4.71)	
Intravenous corticosteroid infusion, *n* (%)			0.016
No	61 (31.12)	14 (16.47)	
Yes	135 (68.88)	71 (83.53)	
IVIG, *n* (%)			0.078
No	189 (96.43)	77 (90.59)	
Yes	7 (3.57)	8 (9.41)	
Replacement of macrolides with tetracyclines/fluoroquinolones before BAL, *n* (%)			0.371
No	52 (26.53)	18 (21.18)	
Yes	144 (73.47)	67 (78.82)	
Chest imaging			
Atelectasis, *n* (%)			<0.001
No	77 (39.29)	10 (11.76)	
Yes	119 (60.71)	75 (88.24)	
Lobar consolidation involving ≥2/3 of a lobe, *n* (%)			<0.001
No	121 (61.73)	28 (32.94)	
Yes	75 (38.27)	57 (67.06)	
Pleural effusion, *n* (%)			<0.001
No	155 (79.08)	45 (52.94)	
Yes	41 (20.92)	40 (47.06)	
Lesions involving ≥2 lobes, *n* (%)			0.098
No	166 (84.69)	65 (76.47)	
Yes	30 (15.31)	20 (23.53)	
Initial bronchoscopic findings			
Bronchial casts involving ≥2 lung lobes, *n* (%)			0.029
No	177 (90.31)	68 (80.00)	
Yes	19 (9.69)	17 (20.00)	
Bilateral lung involvement, *n* (%)			0.758
No	188 (95.92)	81 (95.29)	
Yes	8 (4.08)	4 (4.71)	
Central airway involvement, *n* (%)			—
No	196 (100.00)	85 (100.00)	
Yes	0 (0.00)	0 (0.00)	
Severe airway mucosal injury, *n* (%)			0.666
No	172 (87.76)	73 (85.88)	
Yes	24 (12.24)	12 (14.12)	

WBC, white blood cell count; PLT, platelet count; CRP, C-reactive protein; PCT, procalcitonin; ALT, alanine aminotransferase; AST, aspartate aminotransferase; LDH, lactate dehydrogenase; CK, creatine kinase; CK-MB, creatine kinase-MB isoenzyme; ALB, albumin; IVIG, intravenous immunoglobulin; BAL, bronchoalveolar lavage.

**Table 2:** Interobserver agreement for chest CT findings.

Chest CT findings	*κ* value (95% CI)	*P* value
Atelectasis	0.934 (0.889–0.979)	<0.001
Lobar consolidation involving ≥2/3 of a lobe	0.946 (0.911–0.981)	<0.001
Pleural effusion	0.958 (0.903–1.000)	<0.001
Lesions involving ≥2 lobes	0.921 (0.851–0.991)	<0.001

CT, computed tomography; CI, confidence interval; κ, Cohen's kappa coefficient.

### Predictors of recurrent PB in children

**Figure 2:**
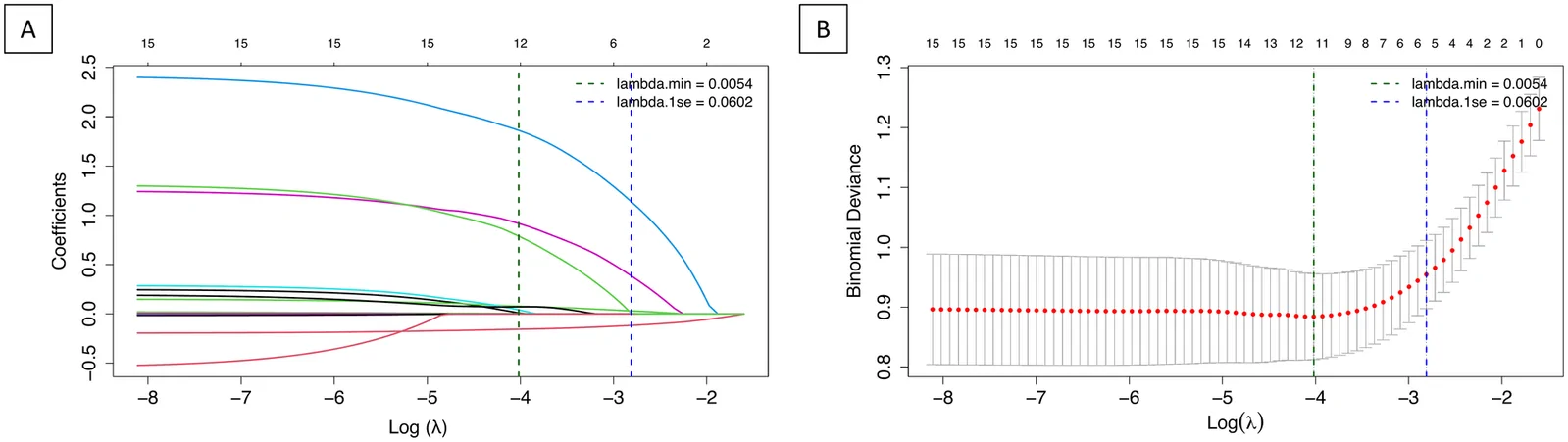
Data statistics and features selection using the LASSO binary logistic regression model. **(A)** LASSO coefficient profiles of the 15 features. A coefficient profile plot was produced against the log(lambda) sequence. **(B)** Optimal parameter (lambda) selection in the LASSO model used fivefold cross-validation via minimum criteria. The partial likelihood deviance (binomial deviance) curve was plotted versus log(lambda).

Based on the between-group comparisons, potential predictive variables were selected for further analysis using LASSO regression. A total of 15 candidate variables were included in the initial analysis, including peak temperature, CRP, PCT, ALT, AST, LDH, CK-MB, ALB, D-dimer, fever persisting for ≥72 h after the first BAL, intravenous corticosteroid therapy, atelectasis, lobar consolidation involving ≥2/3 of a lobe, pleural effusion, and bronchial casts involving ≥2 lung lobes. Using the optimal penalty parameter (*λ*=0.0602) determined by the one-standard-error criterion (*λ*₁se), six variables were retained in the final model: LDH, ALB, D-dimer, fever persisting for ≥72 h after the first BAL, atelectasis, and bronchial casts involving ≥2 lung lobes. These variables were subsequently included as candidate predictors of recurrent PB (Figure 2).

### Prediction model development

**Figure 3:**
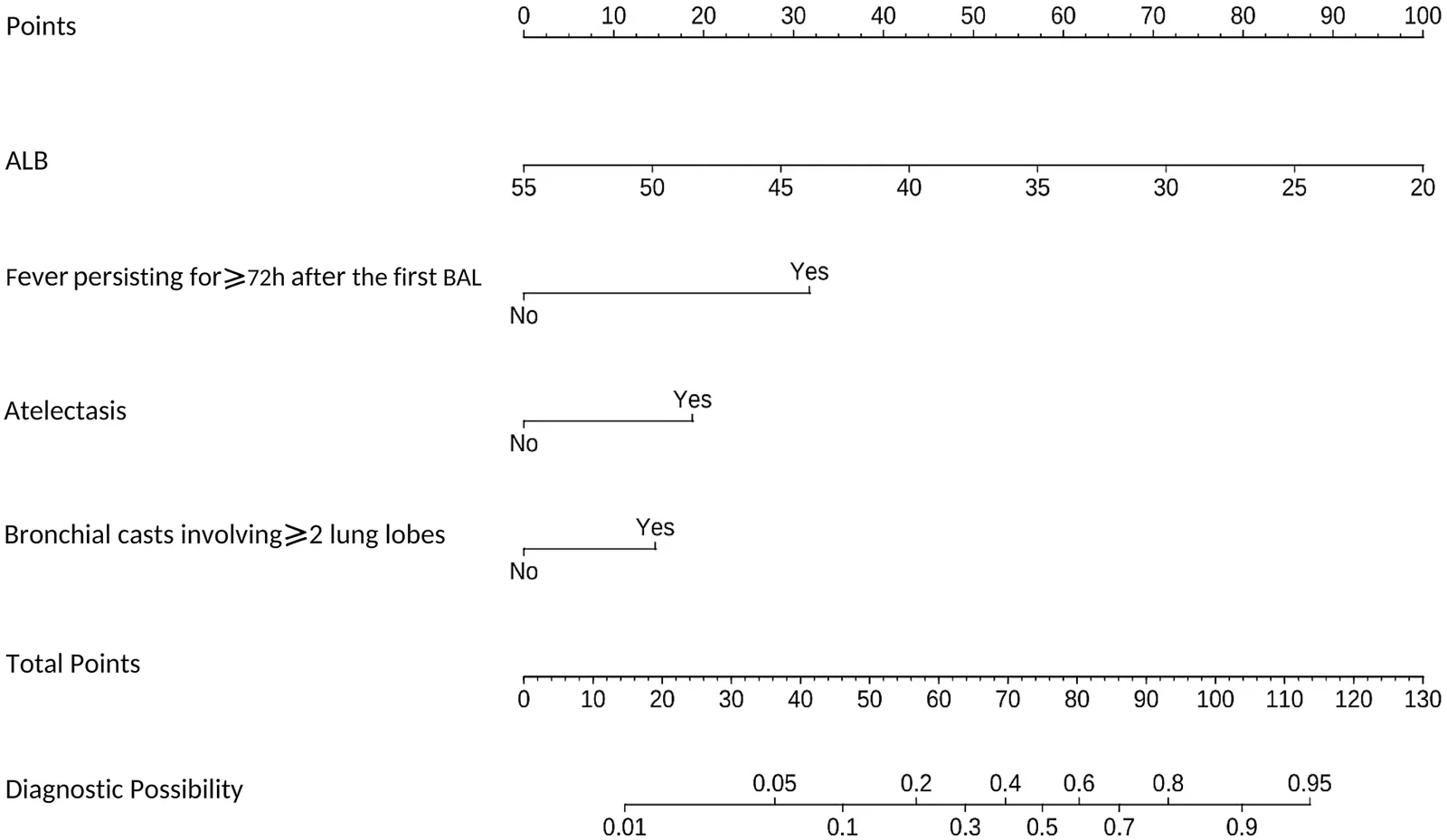
A nomogram predicting recurrence risk of PB in children.

The six variables selected by LASSO regression were subsequently entered into a multivariable logistic regression model. In the multivariable analysis, ALB was inversely associated with recurrent PB (OR = 0.829, 95% CI: 0.769–0.894, *P* < 0.001), whereas fever persisting for ≥72 h after the first BAL (OR = 10.605, 95% CI: 3.711–30.305, *P* < 0.001), atelectasis (OR = 3.825, 95% CI: 1.616–9.054, *P* = 0.002), and bronchial casts involving ≥2 lung lobes (OR = 3.231, 95% CI: 1.281–8.147, *P* = 0.013) were positively associated with recurrent PB. In contrast, D-dimer (OR = 1.164, 95% CI: 0.984–1.378, *P* = 0.076) and LDH (OR = 1.000, 95% CI: 0.998–1.002, *P* = 0.919) were not statistically significant predictors in the multivariable model (Table 3). Accordingly, the four variables that were statistically significant in the multivariable analysis were incorporated into the nomogram (Figure 3).

**Table 3:** Multivariate logistic regression analysis for the independent variables associated recurrent PB in training cohort.

Variables	*β* coefficient	SE	OR	95% CI	Z value	*P* value
ALB	−0.188	0.03857	0.829	0.769–0.894	−4.861	<0.001
Fever persisting for ≥72 h after the first BAL	2.361	0.53569	10.605	3.711–30.305	4.408	<0.001
Atelectasis	1.342	0.43962	3.825	1.616–9.054	3.052	0.002
Bronchial casts involving ≥2 lung lobes	1.173	0.47193	3.231	1.281–8.147	2.485	0.013
D-dimer	0.152	0.08596	1.164	0.984–1.378	1.771	0.076
LDH	0.000	0.00085	1.000	0.998–1.002	0.101	0.919

ALB, albumin; BAL, bronchoalveolar lavage; CI, confidence interval; LDH, lactate dehydrogenase; OR, odds ratio; SE, standard error.

.

To further facilitate the clinical interpretation of ALB, an additional ROC analysis was performed in the training cohort. The optimal cutoff value, determined by maximizing the Youden index, was 41.42 g/L. An ALB level ≤41.42 g/L yielded a sensitivity of 85.88% and a specificity of 58.16% for identifying recurrent PB, with an AUC of 0.776 (Supplementary Figure 1).

### Prediction model validation

**Figure 4:**
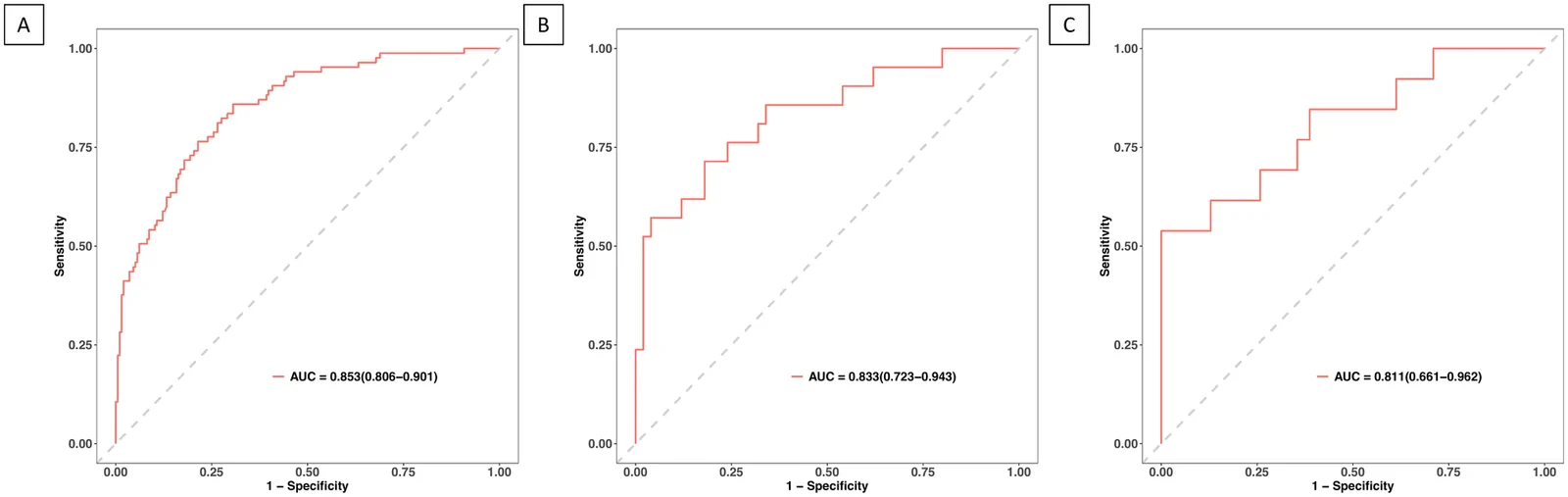
Receiver operating characteristic (ROC) curves of the prediction model. **(A)** ROC curve in the training cohort. **(B)** ROC curve in the internal validation cohort. **(C)** ROC curve in the external validation cohort.

ROC curve analysis demonstrated that the model discriminated recurrent PB from single-episode PB, with an AUC of 0.853 (95% CI: 0.806–0.901) in the training cohort, 0.833 (95% CI: 0.723–0.943) in the internal validation cohort, and 0.811 (95% CI: 0.661–0.962) in the external validation cohort (Figure 4), with similar discrimination across the three cohorts.

**Figure 5:**
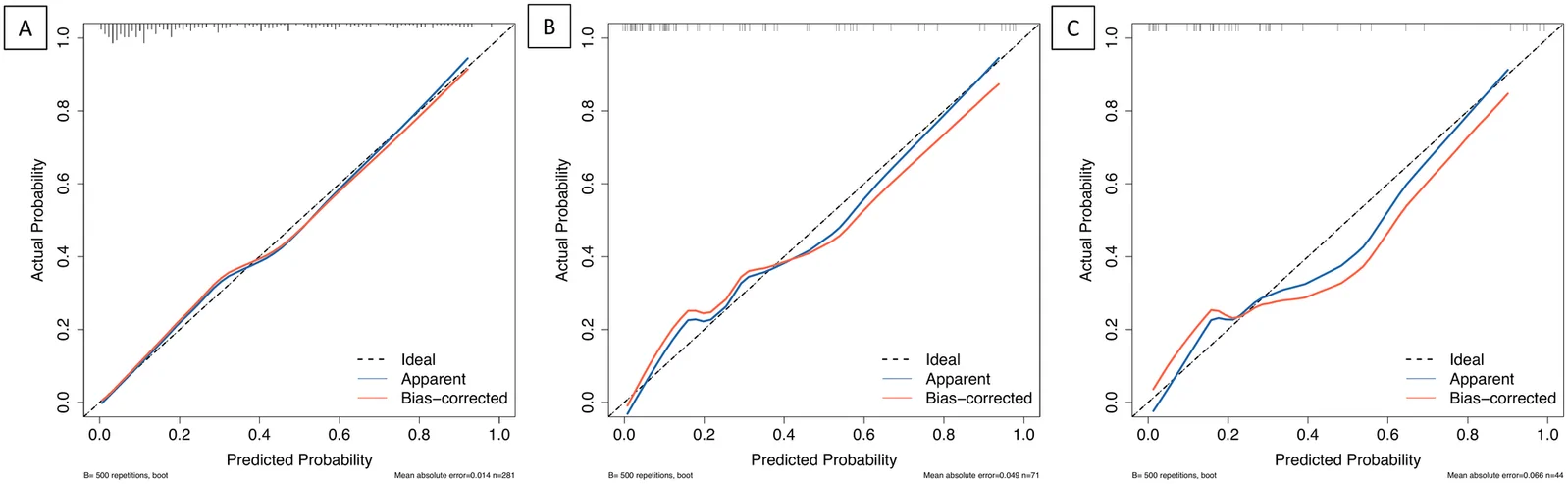
Calibration curve of the risk prediction model for recurrent PB in children. **(A)** Calibration curve in the training cohort. **(B)** Calibration curve in the internal validation cohort. **(C)** Calibration curve in the external validation cohort.

Model calibration was assessed using calibration plots and the Hosmer-Lemeshow goodness-of-fit test. The calibration curves showed agreement between predicted and observed probabilities across the training, internal validation, and external validation cohorts (Figure 5). The Hosmer–Lemeshow goodness-of-fit test also indicated no evidence of poor calibration in the training cohort (*χ*^2^ = 5.1013, *P* = 0.747), internal validation cohort (*χ*^2^ = 7.3472, *P* = 0.499), and external validation cohort (*χ*^2^ = 5.597, *P* = 0.693).

**Figure 6:**
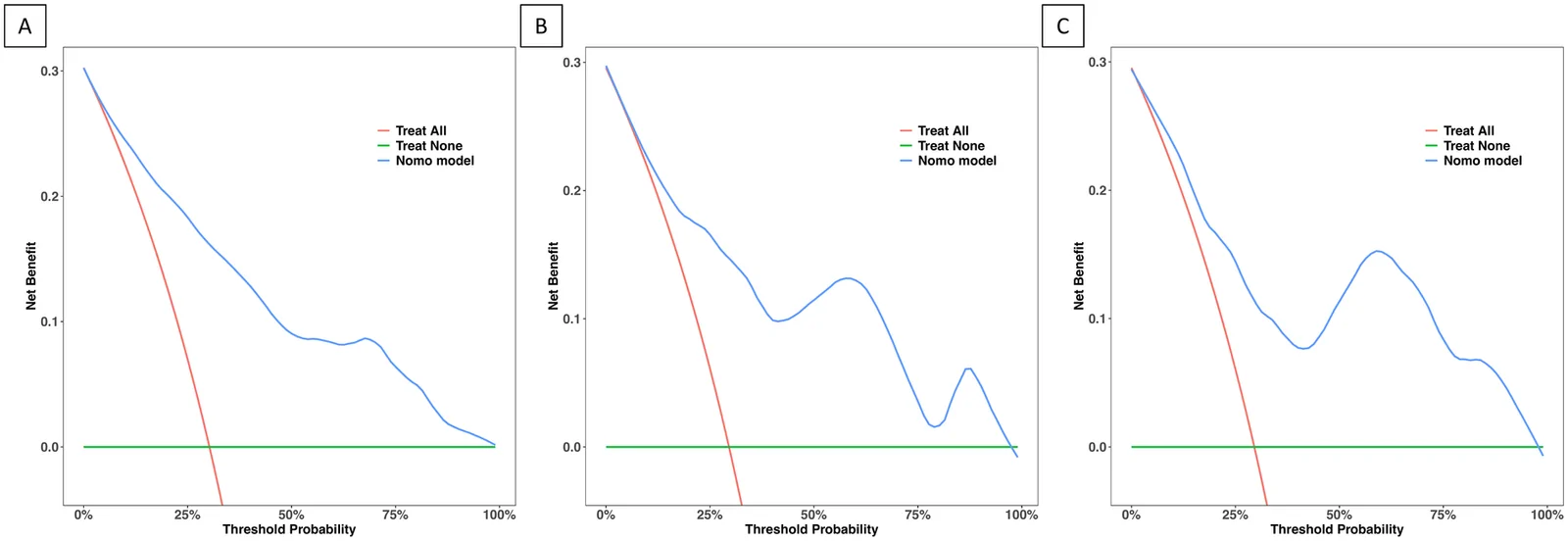
Analysis of decision curves for the nomogram predicting recurrent PB in children. **(A)** Decision curve analysis in the training cohort. **(B)** Decision curve analysis in the internal validation cohort. **(C)** Decision curve analysis in the external validation cohort.

Clinical utility was further examined using DCA. As shown in Figure 6, across a range of threshold probabilities, the nomogram provided a higher net benefit than either the treat-all or treat-none strategies in the training, internal validation, and external validation cohorts.

## Discussion

In this two-center retrospective study of children with MPP complicated by PB, we developed and externally validated an interpretable nomogram for predicting recurrent PB after the initial bronchoscopic removal of plastic casts. The model incorporated four routinely available predictors: ALB, fever persisting for ≥72 h after the first BAL, atelectasis, and bronchial casts involving ≥2 lung lobes. The nomogram showed discrimination across the training, internal validation, and external validation cohorts, with AUCs of 0.853 (95% CI: 0.806–0.901), 0.833 (95% CI: 0.723–0.943), and 0.811 (95% CI: 0.661–0.962), respectively. The model also showed agreement between predicted and observed risks in the three cohorts, and DCA indicated a higher net benefit than the treat-all and treat-none strategies across a range of threshold probabilities. These findings suggest that the nomogram may help stratify the risk of PB recurrence after the first BAL and identify children who may require closer clinical monitoring or reassessment.

Among the predictors retained in the final model, ALB was inversely associated with recurrence risk. Hypoalbuminemia may reflect systemic inflammation and physiological stress in children with severe MPP (13). Beyond its role as an indicator of nutritional status, lower ALB levels have also been associated with inflammation-related endothelial dysfunction and increased vascular permeability (14, 15). These abnormalities may reflect a systemic inflammatory state accompanied by airway wall edema, protein-rich exudation, epithelial injury, and impaired mucociliary clearance, features that have been implicated in bronchial cast formation (16). Thus, lower ALB may serve as a marker of systemic inflammatory burden and disease severity in children with recurrent PB, rather than being interpreted as a causal factor. The optimal cutoff value, determined by maximizing the Youden index, was 41.42 g/L. This value was derived from the present training cohort and represents a data-driven statistical threshold rather than a clinical definition of hypoalbuminemia. Therefore, it should not be interpreted as an albumin reference threshold for clinical diagnosis, and its applicability to other pediatric populations requires validation in independent cohorts.

Persistent fever for≥72 h after the first BAL was another independent predictor of recurrent PB. Clinically, ongoing fever following bronchoscopy may serve as a marker of persistent airway and systemic inflammatory activity despite the initial intervention and subsequent treatment. Previous studies in pediatric MPP-associated PB have similarly reported an association between persistent fever after bronchoscopy and recurrent cast formation (17). In addition, inflammatory profiles in BAL fluid further support persistent fever as a pragmatic surrogate marker of ongoing inflammatory activity (18). Therefore, this readily observable clinical feature may help identify children at increased risk of recurrence and inform subsequent clinical reassessment.

Radiographic findings suggestive of airway obstruction and impaired ventilation are common in children with MPP complicated by PB. Previous studies have reported a high prevalence of atelectasis and pleural effusion among PB cases (6, 16, 19). In our study, atelectasis remained independently associated with recurrent PB. Rather than implying a causal role, atelectasis may serve as a radiographic marker of more pronounced airway obstruction, impaired ventilation, and compromised bronchial drainage. These findings may reflect greater disease severity and persistent airway abnormalities associated with the risk of recurrent cast formation.

Bronchoscopic evidence of cast involvement in ≥2 lung lobes were identified as an independent predictor of PB recurrence. Unlike radiographic findings, this bronchoscopic feature directly reflects the extent of endobronchial obstruction and the cast distribution observed during the initial intervention. In a retrospective study of 107 children with PB, Tian et al. similarly reported that bronchoscopic involvement of ≥2 lung lobes were independently associated with recurrence, with an odds ratio of 3.376, which is consistent with the direction of our findings (20). Previous bronchoscopic and histopathological studies have demonstrated that pediatric PB is characterized by mucosal hyperemia and edema, epithelial shedding, inflammatory cell infiltration, and tenacious branching casts that may extend into multiple lobar or segmental bronchi (6, 21, 22). Multilobar cast involvement may therefore serve as a marker of more extensive airway involvement, epithelial injury, inflammatory exudation, and cast burden. Moreover, casts distributed across multiple lobar or distal segmental bronchi may indicate more extensive endobronchial involvement and greater technical complexity during bronchoscopic removal. Therefore, cast involvement in ≥2 lung lobes may serve as a readily assessable bronchoscopic marker associated with the extent of airway involvement and recurrence risk. This finding may help inform the intensity of post-procedural monitoring and the need for clinical or bronchoscopic reassessment.

Collectively, our model integrates systemic, radiological, and bronchoscopic information using variables that are routinely available in clinical practice (23, 24). From a practical perspective, children with lower ALB levels, fever persisting for ≥72 h after the first BAL, atelectasis, and bronchial casts involving ≥2 lung lobes were more likely to experience recurrent PB in our study population. dentification of these features may support risk-based post-procedural monitoring and clinical reassessment, including consideration of repeat bronchoscopy when clinically indicated. Importantly, given the retrospective observational design of this study, these findings should be interpreted as associations relevant to risk prediction rather than evidence of causal relationships between the identified predictors and PB recurrence (25, 26).

Several limitations should be acknowledged. First, the retrospective design may have introduced selection and outcome ascertainment biases. Repeat bronchoscopy was performed based on clinical indications rather than systematically in all patients; therefore, mild or transient recurrent cast formation in patients who improved without repeat bronchoscopy may have remained undetected. Future prospective studies with standardized follow-up and predefined criteria for repeat bronchoscopy may allow more complete ascertainment of PB recurrence. Second, although independent external validation was performed, the external validation cohort was relatively small, resulting in wide CIs for the AUC and limiting the precision of the validation results. Further validation in larger, geographically diverse, multicenter cohorts is warranted to assess the generalizability and robustness of the model. Third, the clinical impact of incorporating the nomogram into routine practice has not been established and requires prospective evaluation.

## Conclusion

This study developed and externally validated an interpretable nomogram for predicting recurrent PB in children with MPP after the initial bronchoscopic removal of plastic casts. The model incorporates four routinely available predictors: ALB, fever persisting for ≥72 h after the first BAL, atelectasis, and bronchial casts involving ≥2 lung lobes. The nomogram showed consistent discrimination and calibration across the training, internal validation, and external validation cohorts, while DCA indicated a potential net benefit across a range of threshold probabilities. The model may provide a basis for individualized risk stratification and post-procedural monitoring, although its clinical utility has not been established. Further prospective, multicenter studies with larger sample sizes are needed to assess the model's generalizability and determine whether its use can improve clinical decision-making and patient outcomes.

## Data Availability

The raw data supporting the conclusions of this article will be made available by the authors, without undue reservation.
