# Supplementary material for: Development and validation of a nomogram for predicting recurrent plastic bronchitis in children with Mycoplasma pneumoniae pneumonia
Source: Front Pediatr. 2026 Sep 9;14:1959849. doi: 10.3389/fped.2026.1959849 (PMC13597871; doi:10.3389/fped.2026.1959849)
Supplement: Supplementary file 1 [file Supplementaryfile1.docx]

**Supplementary Material**

**Table 1 Missing number of variables**

| **Variables** | **Training cohort(n = 281), n (%)** | **Internal validation cohort(n = 71), n (%)** | **External validation cohort(n = 44), n (%)** |
| --- | --- | --- | --- |
| **Clinical characteristics** |  |  |  |
| Sex | 0 (0.00) | 0 (0.00) | 0 (0.00) |
| Age | 0 (0.00) | 0 (0.00) | 0 (0.00) |
| Duration of fever | 0 (0.00) | 0 (0.00) | 0 (0.00) |
| Fever | 0 (0.00) | 0 (0.00) | 0 (0.00) |
| Peak temperature | 7 (2.49) | 4 (5.63) | 3 (6.82) |
| Cough | 0 (0.00) | 0 (0.00) | 0 (0.00) |
| Dyspnea | 0 (0.00) | 0 (0.00) | 0 (0.00) |
| Hypoxemia | 0 (0.00) | 0 (0.00) | 0 (0.00) |
| **Laboratory tests** |  |  |  |
| WBC | 0 (0.00) | 0 (0.00) | 0 (0.00) |
| PLT | 0 (0.00) | 0 (0.00) | 0 (0.00) |
| CRP | 0 (0.00) | 0 (0.00) | 0 (0.00) |
| PCT | 0 (0.00) | 0 (0.00) | 0 (0.00) |
| ALT | 0 (0.00) | 0 (0.00) | 0 (0.00) |
| AST | 0 (0.00) | 0 (0.00) | 0 (0.00) |
| LDH | 0 (0.00) | 0 (0.00) | 0 (0.00) |
| CK | 0 (0.00) | 0 (0.00) | 0 (0.00) |
| CK-MB | 0 (0.00) | 0 (0.00) | 0 (0.00) |
| ALB | 0 (0.00) | 0 (0.00) | 0 (0.00) |
| D-dimer | 0 (0.00) | 0 (0.00) | 0 (0.00) |
| **Treatment characteristics** |  |  |  |
| Time from onset to the first bronchoscopy | 0 (0.00) | 0 (0.00) | 0 (0.00) |
| Fever persisting for ≥72 h after the first BAL | 0 (0.00) | 0 (0.00) | 0 (0.00) |
| Mechanical ventilation | 0 (0.00) | 0 (0.00) | 0 (0.00) |
| Intravenous corticosteroid infusion | 0 (0.00) | 0 (0.00) | 0 (0.00) |
| IVIG | 0 (0.00) | 0 (0.00) | 0 (0.00) |
| Replacement of macrolides with tetracyclines/fluoroquinolones before BAL | 0 (0.00) | 0 (0.00) | 0 (0.00) |
| **Chest imaging** |  |  |  |
| Atelectasis | 0 (0.00) | 0 (0.00) | 0 (0.00) |
| Lobar consolidation involving ≥2/3 of a lobe | 0 (0.00) | 0 (0.00) | 0 (0.00) |
| Pleural effusion | 0 (0.00) | 0 (0.00) | 0 (0.00) |
| Lesions involving ≥2 lobes | 0 (0.00) | 0 (0.00) | 0 (0.00) |
| **Initial bronchoscopic findings** |  |  |  |
| Bronchial casts involving ≥2 lung lobes | 0 (0.00) | 0 (0.00) | 0 (0.00) |
| Bilateral lung involvement | 0 (0.00) | 0 (0.00) | 0 (0.00) |
| Central airway involvement | 0 (0.00) | 0 (0.00) | 0 (0.00) |
| Severe airway mucosal injury | 0 (0.00) | 0 (0.00) | 0 (0.00) |

**Abbreviations:** WBC, white blood cell count; PLT, platelet count; CRP, C-reactive protein; PCT, procalcitonin; ALT, alanine aminotransferase; AST, aspartate aminotransferase; LDH, lactate dehydrogenase; CK, creatine kinase; CK-MB, creatine kinase-MB isoenzyme; ALB, albumin; IVIG, intravenous immunoglobulin; BAL, bronchoalveolar lavage.

**Table 2 The baseline characteristics were compared among the training, internal validation, and external validation cohorts**

| **Variables** | **Training cohort (n = 281)** | **Internal validation cohort (n = 71)** | **External validation cohort (n = 44)** | **F/χ²/H** | ***P* value** |
| --- | --- | --- | --- | --- | --- |
| Recurrent PB, n (%) |  |  |  | 0.018 | 0.991 |
| No | 196 (69.75) | 50 (70.42) | 31 (70.45) |  |  |
| Yes | 85 (30.25) | 21 (29.58) | 13 (29.55) |  |  |
| **Clinical characteristics** |  |  |  |  |  |
| Sex, n (%) |  |  |  | 0.921 | 0.631 |
| Male | 143 (50.89) | 36 (50.70) | 19 (43.18) |  |  |
| Female | 138 (49.11) | 35 (49.30) | 25 (56.82) |  |  |
| Age, years | 7.66 ± 2.52 | 7.58 ± 2.38 | 7.40 ± 2.48 | 0.225 | 0.799 |
| Duration of fever, days | 8.00 (7.00, 9.50) | 8.00 (7.00, 9.00) | 8.50 (7.00, 9.50) | 2.749 | 0.253 |
| Fever, n (%) |  |  |  | — | — |
| No | 0 (0.00) | 0 (0.00) | 0 (0.00) |  |  |
| Yes | 281 (100.00) | 71 (100.00) | 44 (100.00) |  |  |
| Peak temperature, °C | 39.70 (39.20, 40.00) | 39.70 (39.20, 40.00) | 39.80 (39.30, 40.00) | 0.464 | 0.793 |
| Cough, n (%) |  |  |  | — | — |
| No | 0 (0.00) | 0 (0.00) | 0 (0.00) |  |  |
| Yes | 281 (100.00) | 71 (100.00) | 44 (100.00) |  |  |
| Dyspnea, n (%) |  |  |  | 2.090 | 0.352 |
| No | 257 (91.46) | 61 (85.92) | 39 (88.64) |  |  |
| Yes | 24 (8.54) | 10 (14.08) | 5 (11.36) |  |  |
| Hypoxemia, n (%) |  |  |  | 0.492 | 0.782 |
| No | 250 (88.97) | 65 (91.55) | 40 (90.91) |  |  |
| Yes | 31 (11.03) | 6 (8.45) | 4 (9.09) |  |  |
| **Laboratory tests** |  |  |  |  |  |
| WBC, 10⁹/L | 7.95 (6.44, 9.79) | 7.21 (5.62, 9.13) | 7.57 (5.98, 8.88) | 2.939 | 0.23 |
| PLT, 10⁹/L | 260.00 (217.00, 318.00) | 278.00 (216.00, 327.00) | 256.00 (222.00, 288.00) | 2.539 | 0.281 |
| CRP, mg/L | 20.40 (11.31, 40.09) | 21.28 (14.03, 43.38) | 27.28 (13.88, 46.88) | 3.160 | 0.206 |
| PCT, ng/mL | 0.24 (0.15, 0.41) | 0.25 (0.18, 0.52) | 0.22 (0.15, 0.41) | 2.511 | 0.285 |
| ALT, U/L | 15.84 (10.42, 21.69) | 14.44 (8.39, 19.40) | 16.64 (11.60, 22.25) | 1.951 | 0.377 |
| AST, U/L | 33.99 (24.28, 46.82) | 32.97 (23.77, 44.58) | 32.41 (21.06, 40.50) | 0.696 | 0.706 |
| LDH, U/L | 386.18 (289.96, 493.62) | 351.45 (256.00, 438.96) | 400.78 (299.21, 468.51) | 3.321 | 0.19 |
| CK, U/L | 88.37 (52.91, 155.87) | 91.36 (54.33, 150.56) | 71.96 (43.24, 151.10) | 0.618 | 0.734 |
| CK-MB, U/L | 22.72 (14.92, 31.99) | 21.12 (13.19, 30.93) | 22.78 (14.85, 34.12) | 0.570 | 0.752 |
| ALB, g/L | 40.94 ± 5.78 | 40.50 ± 5.49 | 41.76 ± 5.27 | 0.676 | 0.509 |
| D-dimer, mg/L | 1.16 (0.61, 2.34) | 0.95 (0.52, 2.32) | 1.54 (0.83, 2.64) | 3.430 | 0.18 |
| **Treatment characteristics** |  |  |  |  |  |
| Time from onset to the first bronchoscopy, days | 9.00 (7.00, 10.00) | 8.50 (7.00, 10.00) | 9.00 (7.00, 10.00) | 2.027 | 0.363 |
| Fever persisting for ≥72 h after the first BAL, n (%) |  |  |  | 3.035 | 0.219 |
| No | 253 (90.04) | 63 (88.73) | 43 (97.73) |  |  |
| Yes | 28 (9.96) | 8 (11.27) | 1 (2.27) |  |  |
| Mechanical ventilation, n (%) |  |  |  | — | 0.712 |
| No | 274 (97.51) | 68 (95.77) | 43 (97.73) |  |  |
| Yes | 7 (2.49) | 3 (4.23) | 1 (2.27) |  |  |
| Intravenous corticosteroid infusion, n (%) |  |  |  | 1.989 | 0.37 |
| No | 75 (26.69) | 18 (25.35) | 16 (36.36) |  |  |
| Yes | 206 (73.31) | 53 (74.65) | 28 (63.64) |  |  |
| IVIG, n (%) |  |  |  | — | 0.519 |
| No | 266 (94.66) | 68 (95.77) | 40 (90.91) |  |  |
| Yes | 15 (5.34) | 3 (4.23) | 4 (9.09) |  |  |
| Replacement of macrolides with tetracyclines/fluoroquinolones before BAL, n (%) |  |  |  | 0.374 | 0.829 |
| No | 70 (24.91) | 20 (28.17) | 12 (27.27) |  |  |
| Yes | 211 (75.09) | 51 (71.83) | 32 (72.73) |  |  |
| **Chest imaging** |  |  |  |  |  |
| Atelectasis, n (%) |  |  |  | 2.534 | 0.282 |
| No | 87 (30.96) | 29 (40.85) | 14 (31.82) |  |  |
| Yes | 194 (69.04) | 42 (59.15) | 30 (68.18) |  |  |
| Lobar consolidation involving ≥2/3 of a lobe, n (%) |  |  |  | 0.860 | 0.651 |
| No | 149 (53.02) | 42 (59.15) | 24 (54.55) |  |  |
| Yes | 132 (46.98) | 29 (40.85) | 20 (45.45) |  |  |
| Pleural effusion, n (%) |  |  |  | 1.644 | 0.44 |
| No | 200 (71.17) | 45 (63.38) | 31 (70.45) |  |  |
| Yes | 81 (28.83) | 26 (36.62) | 13 (29.55) |  |  |
| Lesions involving ≥2 lobes, n (%) |  |  |  | 0.516 | 0.773 |
| No | 231 (82.21) | 56 (78.87) | 35 (79.55) |  |  |
| Yes | 50 (17.79) | 15 (21.13) | 9 (20.45) |  |  |
| **Initial bronchoscopic findings** |  |  |  |  |  |
| Bronchial casts involving ≥2 lung lobes, n (%) |  |  |  | 2.287 | 0.319 |
| No | 245 (87.19) | 57 (80.28) | 37 (84.09) |  |  |
| Yes | 36 (12.81) | 14 (19.72) | 7 (15.91) |  |  |
| Bilateral lung involvement, n (%) |  |  |  | — | 0.589 |
| No | 269 (95.73) | 69 (97.18) | 41 (93.18) |  |  |
| Yes | 12 (4.27) | 2 (2.82) | 3 (6.82) |  |  |
| Central airway involvement, n (%) |  |  |  | — | — |
| No | 281 (100.00) | 71 (100.00) | 44 (100.00) |  |  |
| Yes | 0 (0.00) | 0 (0.00) | 0 (0.00) |  |  |
| Severe airway mucosal injury, n (%) |  |  |  | 0.644 | 0.725 |
| No | 245 (87.19) | 61 (85.92) | 40 (90.91) |  |  |
| Yes | 36 (12.81) | 10 (14.08) | 4 (9.09) |  |  |

**Abbreviations:** WBC, white blood cell count; PLT, platelet count; CRP, C-reactive protein; PCT, procalcitonin; ALT, alanine aminotransferase; AST, aspartate aminotransferase; LDH, lactate dehydrogenase; CK, creatine kinase; CK-MB, creatine kinase-MB isoenzyme; ALB, albumin; IVIG, intravenous immunoglobulin; BAL, bronchoalveolar lavage.

**Note:** Data are presented as mean ± standard deviation (SD), median (interquartile range [IQR]), or n (%), as appropriate. *P* values represent overall comparisons across the three cohorts. “—” indicates that no F/χ²/H statistic was available for exact tests or that inferential testing was not performed because of no variation across cohorts.

**Fig. 1 Receiver operating characteristic (ROC) curve of serum albumin (ALB) for identifying recurrent plastic bronchitis (PB) in the training cohort.**The optimal cutoff value of ALB was 41.42 g/L, as determined by the maximum Youden index, with a sensitivity of 85.88%, a specificity of 58.16%, and an area under the curve (AUC) of 0.776.
